# Noninvasive measurement of dynamic brain signals using light penetrating the brain

**DOI:** 10.1371/journal.pone.0192095

**Published:** 2018-01-31

**Authors:** Osamu Hiwaki, Hideki Miyaguchi

**Affiliations:** 1 Graduate School of Information Sciences, Hiroshima City University, Hiroshima, Japan; 2 Graduate School of Biomedical and Health Sciences, Hiroshima University, Hiroshima, Japan; Tokai University, JAPAN

## Abstract

Conventional techniques for the noninvasive measurement of brain activity involve critical limitations in spatial or temporal resolution. Here, we propose the method for noninvasive brain function measurement with high spatiotemporal resolution using optical signals. We verified that diffused near-infrared light penetrating through the upper jaw and into the skull, which we term as optoencephalography (OEG), leads to the detection of dynamic brain signals that vary concurrently with the electrophysiological neural activity. We measured the OEG signals following the stimulation of the median nerve in common marmosets. The OEG signal response was tightly coupled with the electrophysiological response represented by the somatosensory evoked potential (SSEP). The OEG measurement is also shown to offer rather clear discrimination of brain signals.

## Introduction

The development of noninvasive techniques to allow the measurement of brain activity is imperative for revealing how the human brain functions. Conventional techniques for noninvasive measurement of brain activity such as functional magnetic resonance imaging (fMRI), near-infrared spectroscopy (NIRS), magnetoencephalography (MEG), and electroencephalography (EEG), are burdened by critical limitations in spatial or temporal resolution [1]. In order to improve the accuracy of spatiotemporal estimates of dynamic brain activity, information from multiple conventional techniques can be combined [2]. The need for techniques that allow for noninvasive measurement of brain activity accompanied by high spatial and temporal resolution is apparent. NIRS, which uses near-infrared light similar to that in this study, has been used to measure cerebral activity [3]. The signals detected using NIRS originate from changes in the concentration of oxyhemoglobin and deoxy-hemoglobin due to vascular changes in the cortex. However, following an electrophysiological response, the hemodynamic response is delayed by several seconds. Although the event-related optical signal has a latency of up to hundreds of milliseconds introduced by the phase lag in the evoked scattering changes accessible with a conventional NIRS [4,5], the reliability of such a signal remains a matter of debate [6,7]. Furthermore, when using a conventional NIRS, the distance between the source and the detector located on the skull is ranges from 20–40 mm. As the light diffuses in all directions inside the head both before and after passing through the brain tissue, the distribution of the light is rather complex in the conventional NIRS. This means that it is difficult to achieve high spatial resolution by the conventional NIRS [8]. In this study, we demonstrate a noninvasive brain function measurement with high spatiotemporal resolution, using near-infrared light to penetrate through the upper jaw and into the skull, which we term as optoencephalography (OEG).

## Material and methods

### Subject

In this study, three healthy common marmosets (weight 290–360 g, CLEA Japan, Osaka, Japan) were assessed. The marmosets were placed in a regular day-night cycle and had free access to food and water. This study was approved by the Ethics Committee for Experimental Animals of Hiroshima University. The marmosets were treated according to the guidelines stipulated by the Institutional Animal Care and Use Committee. The animals were diverted to other studies after the end of the study.

### Somatosensory stimulation

To induce a rapid inhalation anesthesia, a concentration of 2.5% of isoflurane (Intervet, Tokyo, Japan) (3 l/min air) for about 5 min. Then, the concentration of isoflurane was reduced and maintained at 1.0% for the continuation of the experiment. We monitored blood oxygen saturation level (SpO_2_) and heart rate by a pulse oximeter during the OEG or SSEP measurements. An electrical stimulation was applied to the median nerve of the left wrist using 1.5 mm long needle electrodes. The electric current stimulation consisted of a monophasic square wave pulse (100 μs in duration) applied just above the motor threshold to elicit a slight but visible twitch of the thumb every 1.7 s.

### Recording of OEG and SSEP

**Fig 1:**
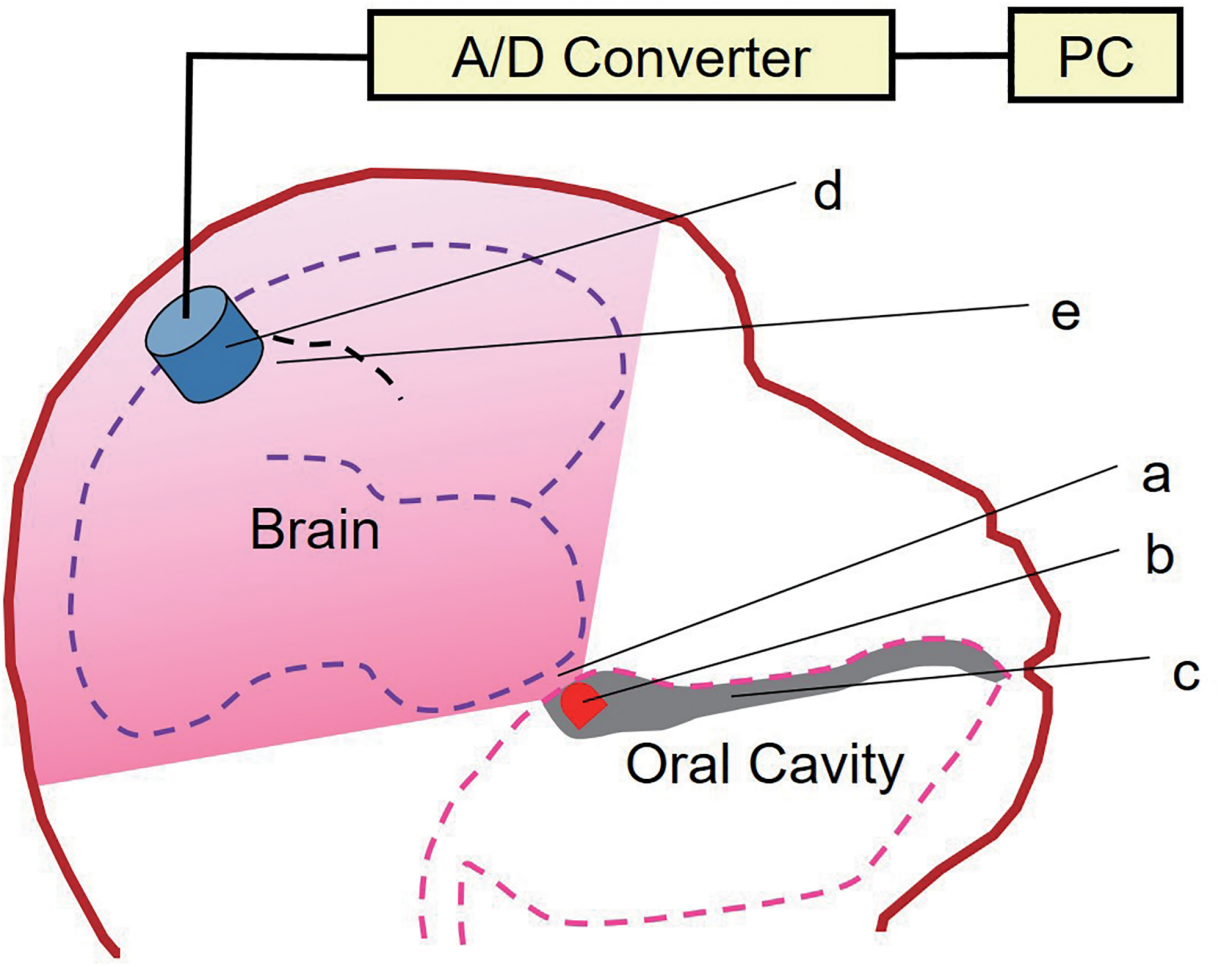
Schematic drawing of the optoencephalography (OEG) measurement system. Near-infrared light (a) was emitted from a light-emitting diode (LED) (b) located on the palate at the upper jaw. The LED was embedded in the polyethylene resin shaped like a mouth-guard on the palate of the upper jaw (c). The near-infrared light penetrating the brain was detected on the scalp using a photo-sensor module (d). The optical signal measured with the photo-sensor module was sampled with an A/D converter. The photo-sensor was placed on the scalp and aimed at the hand region of the postcentral gyrus of the cortex (corresponding to the S1 primary somatosensory cortex) (e).

The marmoset’s head was shaved. During the recording sessions, the marmoset was placed in a wooden restraining chair. For OEG recording, an LED (SFH 4713A, OSRAM Opto Semiconductors, Regensburg, Germany) emitting 850 nm near-infrared light was fixed on the palate in the mouth. The LED was embedded in polyethylene resin, shaped in the form of a mouth-guard, and fitted onto the palate of the upper jaw, as shown in Fig 1. The light power of the LED was adjusted with an LED driver just above the threshold to measure OEG signals. The near-infrared light penetrating the head was detected on the scalp using a photo-sensor module (H10722-20, Hamamatsu Photonics, Hamamatsu, Japan) which is sensitive to signals of up to 920 nm. The center of the photo-sensor window (a 10 mm diameter circle) was placed 7 mm to the right of the skull vertex (R7, 0). This is located above the hand region of the primary somatosensory cortex of a marmoset [9]. The OEG signal was expressed as the average of 300 values of Δ*I*/*I* as in the previous studies on optical measurement of neuronal signals [10–13]; the average optical signal during a 125 ms pre-stimulus period was defined as *I*; and the difference between the measured optical signal and *I* was defined as Δ*I*. Δ*I/I* was sampled at 5 kHz using a 16-bit A/D converter beginning 250 ms before stimulation and ending 1000 ms after the stimulation. In order to evaluate the validity of the OEG signals, SSEP recording was conducted. In the SSEP recording, the center point of a sintered Ag/AgCl recording electrode of 10 mm diameter was placed at (R7, 0), e.g. at the same position as the photo-sensor in the OEG measurement. The reference and ground electrodes were placed at the left and right earlobes, respectively. EEG recordings were taken using a DC amplifier with a low-pass filter at 500 Hz and sampled with a 16 bit A/D converter, similar to that used in the OEG recording. The average of 300 EEG signals was calculated to resolve the SSEP. We measured the OEG signals at four points surrounding the position (R7, 0) in order to evaluate the spatial resolution of OEG. The positions for OEG measurement were: vertex (0, 0); 14 mm right from vertex (R14, 0); 7 mm right and 7 mm anterior from vertex (R7, A7); 7 mm right and 7 mm posterior from vertex (R7, P7) in addition to (R7, 0) as shown in Fig 3(a). To obtain stable SSEP or OEG signals, the signals observed between 0.5 h to 1.5 h after the beginning of inhalation anesthesia were used for analysis. We obtained multiple data sets from OEG and SSEP measurements in order to verify their reproducibility. The cross-correlation coefficient between the grand averages of the OEG and SSEP across all subjects was calculated using MATLAB (MathWorks, Massachusetts, USA).

## Results

**Fig 2:**
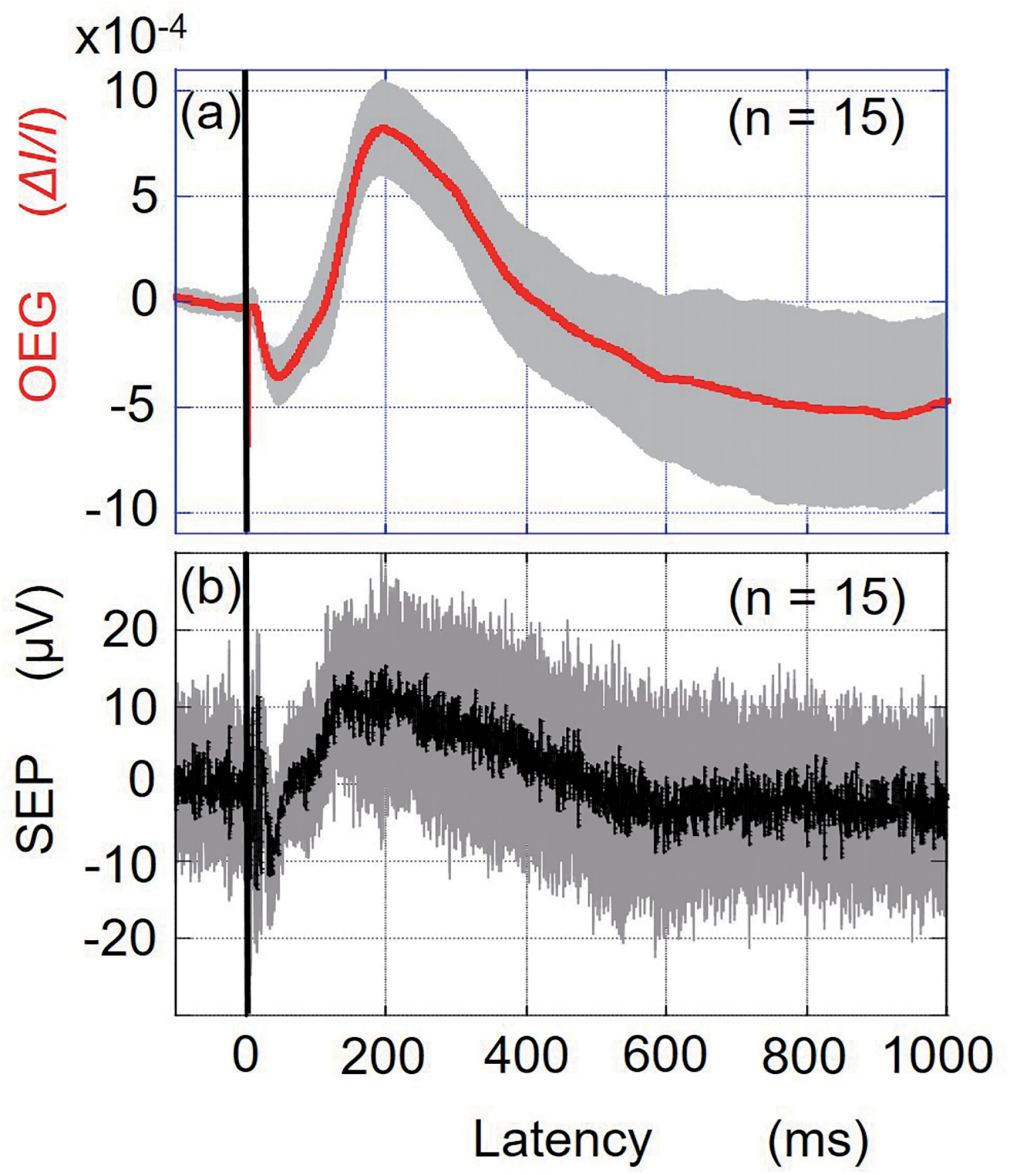
Optoencephalography (OEG) (a) and somatosensory evoked potential (SSEP) (b) responses. The red and black lines represent the averages of the OEG measurements (15 trials across all subjects) and SSEP measurements (15trials across all subjects) respectively. The grey lines indicate the standard deviations at each sampling time. Each component of the OEG or SSEP consisted of an average of 300 trials.

**Fig 3:**
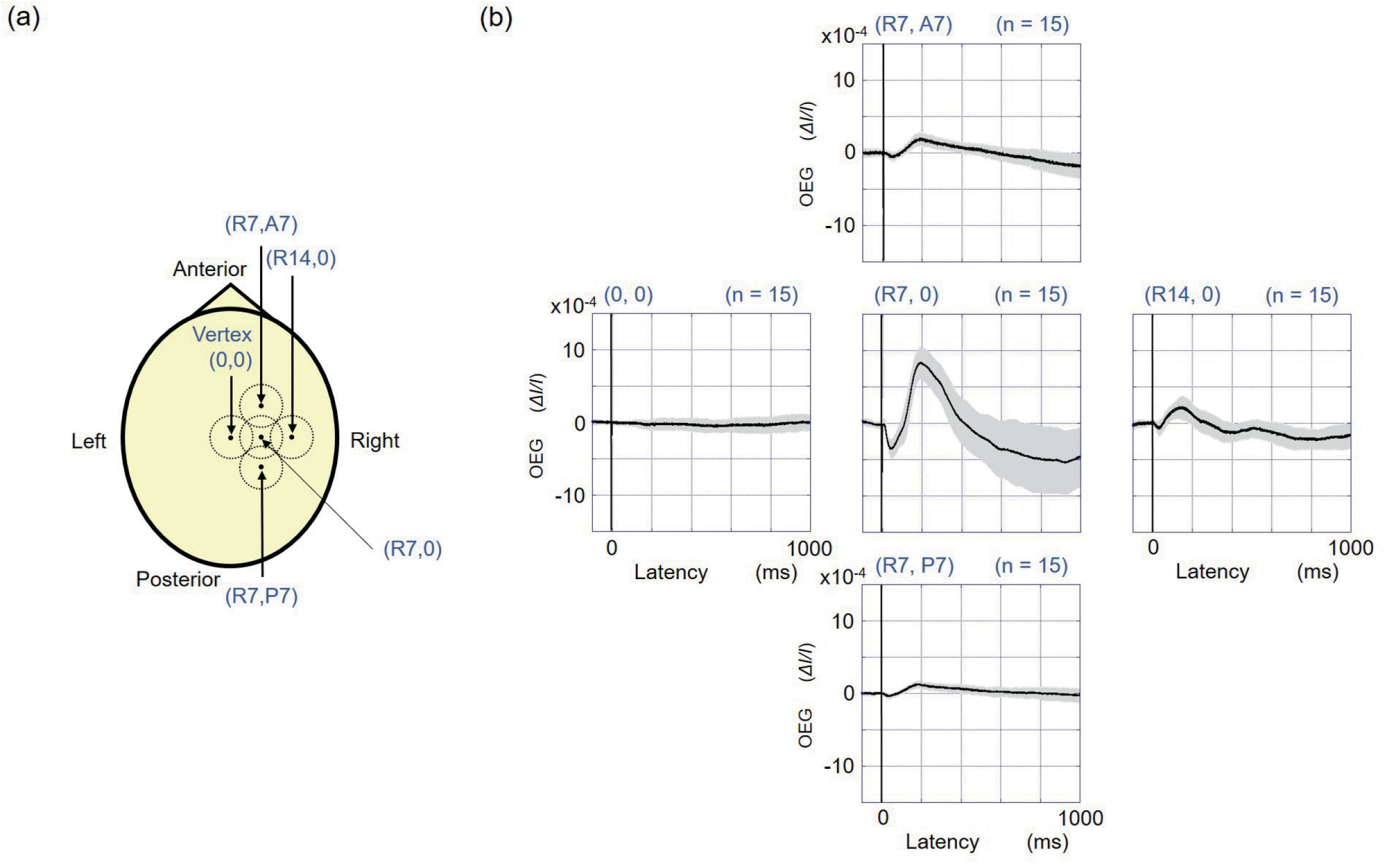
Locations of photo-sensors (a) and observed optoencephalography (OEG) signals (b). The locations of photo-sensors are vertex (0, 0); 14 mm right from vertex (R14, 0); 7 mm right and 7 mm anterior from vertex (R7, A7); 7 mm right and 7 mm posterior from vertex (R7, P7); in addition to (R7, 0). Dotted circles in locations of photo-sensors represent the window area of the photo-sensor. In the OEG signals, the black lines represent the averages of the OEG, and the grey lines indicate the standard deviations at each sampling time (15trials across all subjects).

The OEG response measured at (R7, 0), which is 7 mm to the right of the skull vertex, is presented in Fig 2(a). The OEG response began to decrease with a latency of 11.8 ± 1.4 ms (mean ± SD, 15 trials across all subjects). The negative peak latency and positive peak latency of the OEG response were 43.9 ± 5.1 ms and 192.0 ± 12.3 ms, respectively. Fig 2(b) shows the time course of the somatosensory evoked potential (SSEP). The negative peak after the fast response was 37.7 ± 1.5 ms (mean ± SD, 15 trials across all subjects), followed by a positive peak at 194.3 ms ± 17.8 ms. The negative peak noted at approximately 40 ms and the positive peak at approximately 190 ms of the OEG response corresponded with those recorded for the SSEP response. The cross-correlation coefficient between the averages of the OEG and SSEP following stimulation was 0.72, which denotes high similarity between the OEG and SSEP. As shown in Fig 3(a), the OEGs were measured at four additional points around position (R7, 0), in order to evaluate the spatial discrimination of OEG. Fig 3(b) shows that the amplitudes of the OEG responses measured at four points located 7 mm from (R7, 0) were less than halved from the original measurement at (R7, 0). Especially, the OEG signal measured at the vortex was very little. A two-sample *t*-test shows that the OEG amplitudes of the negative and positive peaks measured at the four points: (0, 0), (R14, 0), (R7, A7) and (R7, P7) shown in Fig 3(b) are significantly different (P < 0.01) from those at (R7, 0).

## Discussion

Our results demonstrate that the OEG signal response is tightly coupled to the electrophysiological response, following a sensory stimulation of the peripheral nerve. The high cross-correlation coefficient of 0.72 between the OEG and SSEP indicates a strong relationship between the OEG signal and the electrophysiological neural activity in the brain. Our findings in this study revolutionize the measurement of brain dynamic function, which has been difficult to achieve using conventional methods such as NIRS, fMRI, MEG and EEG. These conventional methods are constrained by limitations in temporal or spatial resolution. The signal for neural activity measured by NIRS or fMRI is based on the hemodynamic response, and therefore, the attainable temporal resolution is in the range of a few seconds. The measurement with EEG/MEG has a temporal resolution in the order of milliseconds; however, the problem of estimating the current sources in EEG/MEG recordings is fundamentally difficult and without solution to date [14]. The studies using cell cultures or tissue preparations have shown changes in the back-scattered or birefringence light following nerve stimulation [10–13]. The response in the OEG immediately after the stimulation observed in this study is in line with the studies which observed optical signals associated with electrophysiological activation in isolated nerves [12,13]. The light changes that are observed in cell cultures or tissue preparations are thought to be due to conformational changes and the swelling of the nerve cells [13]. Therefore, the OEG response observed in this study is likely caused by the optical response to conformational changes and swelling of the nerve cells in the brain. In conventional NIRS, both the light source and the detector are located on the skull. This means that the positional relationship of the light source and the detector in conventional NIRS is not suitable for detecting the neural activity with back-scattered or birefringence light of the cortical nerve cells. As shown in Fig 3, the smaller OEG signals surrounding the position where a large OEG signal was observed. The OEG signal measured at the vertex was very little. This indicates that the OEG signal reflects cortical activity with high spatial resolution, since cortical matter does not exist in the longitudinal fissure of the cerebrum beneath the vertex. This observation also indicates that the OEG signal does not arise from artifact like body movement. The estimation of the active cortical locations with OEG is supposed to be simpler and more precise than by use of EEG/MEG. The measured signals in EEG/MEG are generated by neuronal currents in the cortex within which the neuronal population is synchronously active. The activity of distantly placed sources in the brain can be measured on same sensors in EEG/MEG due to diffusive effects of fields and potentials [15]. In such conditions, it is difficult to clarify the location of active cortical area. The spatial resolution of conventional SSEP measurement is inferior to that seen in the OEG response observed in this study. The SSEP signal is distributed across a large area of the scalp which includes the skull vertex [16]. Although OEG was not measured simultaneously at different positions in this study, our results indicate that it is possible to measure brain signals with both high temporal and spatial resolution by simultaneously measuring OEG responses on the skull. In the OEG, the neuronal signals caused by conformational changes and swelling of the nerve cells in the cortex at the outermost layer of the brain can be most measured by the nearby sensors on the scalp. The active cortical location can be estimated as the point on the cortical surface just beneath the sensor detecting the large OEG signal. The optical method proposed in this study is suitable for the brain-machine interface (BMI) or brain-computer interface (BCI) because it is tolerant to electromagnetic artifact that contaminates EEG/MEG. Although a human head is larger than a head of a common marmoset, the OEG signal observed in this study is clear enough to expect availability of the method for the human. Moreover, our method is easy to use and the cost for introduction and maintenance is lower than that of conventional methods.

## Conclusion

In this study, we proposed the noninvasive method to measure the brain signals using diffused near-infrared light penetrating the head from upper jaw to scalp, which is denoted as OEG. In order to verify the validity of OEG, we observed OEG response following the somatosensory stimulation in common marmosets. The results show that OEG signal response is tightly coupled with the electrophysiological response represented by the SSEP. We also confirmed that the OEG measurement offer rather clear discrimination of brain signals.
